# The Features of COVID-19’s Course and the Efficacy of the Gam-COVID-Vac Vaccine in Patients with Paroxysmal Nocturnal Hemoglobinuria

**DOI:** 10.3390/hematolrep15030052

**Published:** 2023-09-01

**Authors:** Vadim Ptushkin, Evgeniya Arshanskaya, Olga Vinogradova, Dmitry Kudlay, Eugene Nikitin

**Affiliations:** 1 Moscow City Hematology Center, S.P. Botkin City Clinical Hospital, Moscow 125284, Russia; 2 Department of Oncology, Hematology and Radiation Therapy, Pirogov Russian National Research Medical University, Moscow 117997, Russia; 3 Department of Pharmacology, Pharmacy Institute, I.M. Sechenov First Moscow State Medical University (Sechenov University), Moscow 119991, Russia; 4 Hematology and Transfusiology Department, Russian Medical Academy of Continuous Professional Education, Moscow 125993, Russia

**Keywords:** paroxysmal nocturnal hemoglobinuria, COVID-19, Gam-COVID-Vac, SARS-CoV-2, anti-SARS-CoV-2 IgG, eculizumab

## Abstract

COVID-19 and other infectious diseases can exacerbate the course of paroxysmal nocturnal hemoglobinuria (PNH). The efficacy and safety of the Gam-COVID-Vac vaccine in patients with PNH has not been adequately studied. A retrospective, observational, cohort, non-comparative study was performed to assess the course of COVID-19 as well as the safety and efficacy of the Gam-COVID-Vac (Sputnik V) vaccine in patients with paroxysmal nocturnal hemoglobinuria (PNH). The study included data from 52 patients with PNH aged 18 to 75 years, 38 of whom received background therapy with eculizumab (Elizaria^®^) between March 2020 and January 2022. COVID-19 was diagnosed according to the results of PCR testing. The patients were divided into two groups for comparison of the incidence of COVID-19. Group 1 included non-vaccinated patients with PNH, and Group 2 included patients vaccinated prior to the onset of COVID-19. According to vaccination, patients were subdivided into non-vaccinated and vaccinated groups without signs of previous COVID-19 at the beginning of the analyzed period, and patients vaccinated half a year or more after recovery from COVID-19. Testing for anti-SARS-CoV-2 IgG levels was carried out in patients with PNH in the year after their COVID-19. Tests for anti-SARS-CoV-2 RBD IgG levels were performed on vaccinated patients. In total, 28 (53.8%) of the enrolled patients had COVID-19, including asymptomatic forms in 7 (25%) and mild forms in 16 (57%) patients. A total of 22 (42.3%) patients were fully vaccinated with Gam-COVID-Vac, of which 13 (25%) patients were vaccinated without the signs of previous SARS-CoV-2infection, and 9 (17.3%) patients were vaccinated after COVID-19. The number of patients who had COVID-19 was about two times higher in Group 1 (non-vaccinated; 24) (61.5%), whereas in Group 2 (vaccinated), the number of patients with COVID-19 was only 4 (30.8%). The proportion and number of patients who did not have COVID-19 was higher in the group of vaccinated patients (9; 69.2%) than in the group of non-vaccinated patients (15; 38.5%) (*p* = 0.054). In patients who had been infected with COVID-19, maximum concentrations of anti-SARS-CoV-2 IgG were observed 2–3 months after the acute infection phase, followed by a gradual decline by month 9–10. The mean *RBD* IgG concentration was higher in the group of patients who had been infected by COVID-19 than in the group of patients without COVID-19 (*p* = 0.047). Therapy type, including eculizumab, did not have a significant impact on RBD IgG titers (*p* > 0.05). Hospitalization was required in five (18%) patients, all of whom had breakthrough hemolysis and severe lung damage on CT scans. After the first dose, adverse events (AEs) were reported in 41% of the patients (body temperature increased in 18%; headache in 13.6%; and pain in joints in 4.5%; colitis exacerbation was observed in 4.5%). After the second dose, no AEs were reported. The performed study suggests the possible efficacy and demonstrates the safety of Gam-COVID-Vac (Sputnik V) for the prophylaxis of COVID-19 in patients with PNH who experience immunosuppression due to target therapy.

## 1. Introduction

Paroxysmal nocturnal hemoglobinuria (PNH) is a rare (orphan), acquired clonal blood disorder, characterized by bone marrow impairment, chronic complement-mediated hemolysis, and thrombosis [1]. The development of PNH is genetically caused by somatic mutation in the PIGA (phosphatidylinositol glycan anchor biosynthesis class A) gene, resulting in GPI (glycosylphosphatidylinisotol) protein deficiency and the poor binding of CD55 and CD59 to the cell membrane [1,2]. PNH is an extremely rare disease. According to the international registry of PNH, initiated in 2003, the incidence of PNH is 1–1.5 per 1,000,000 population per year [3]. The prevalence is 1.59 per 100,000 people [4]. In Russia, the current prevalence of PNH is about 5.4 per 1,000,000 people; in Moscow, it is 4.1 per 1,000,000 people. Flow cytometry with individual monoclonal antibodies (anti-CD55, anti-CD59, anti-CD14, or FLAER) is widely used in PNH diagnosis and monitoring, including in patients after infection [5].

In recent years, treatment with eculizumab (a humanized monoclonal antibody that binds to complement component C5) in patients with PNH has been successful. Eculizumab, by blocking the final stage of complement activation and intravascular hemolysis, significantly reduces the incidence of complications, and improves the patients’ quality of life [6]. In 2011, eculizumab was authorized in Russia under the name Soliris. In March 2019, the first biosimilar of eculizumab, Elizaria^®^, was authorized, and began to be used [7,8,9].

Over the past few years, the issue of COVID-19 in patients with PNH and the impact of vaccination against this infection on the course of PNH have become even more urgent issues due to the COVID-19 pandemic. The data on these issues are represented by single case reports. For example, an Italian study of COVID-19 in patients with PNH showed that infection was asymptomatic in two out of four patients, and mild in the other two patients. One case of breakthrough hemolysis was reported in a patient who was not receiving target therapy. The incidence of SARS-CoV-2 infection in patients with PNH was not greater compared with the general population [10]. Fattizzo B. et al. (2021) described 19 cases of hemolysis of differing severity in patients with PNH, who were predominantly on anti-complement treatment. In 89.4% of patients, mild and moderate-to-severe forms of COVID-19 were reported; two patients required hospitalization in the intensive care unit, and one patient died of severe respiratory failure [11].

Gerber G.F. et al. (2021) described a series of clinical complications after vaccination against SARS-CoV-2 with messenger RNA (mRNA)-based vaccines, such as Pfizer-BioNTech and Moderna, in patients with PNH. Serious adverse events (SAEs) after vaccination were reported in four patients; two patients tolerated vaccination without significant AEs or hemolysis. At the time of vaccination, the patients’ age was 25 to 63 years; all patients had a pathological clone on PNH granulocytes ≥80%, and they had not received hemosubstitution treatment within the last year. Adverse reactions occurred from day 1 to day 5, and lasted 1–6 days. Fever was reported in four out of six patients, severe hemolysis with hemoglobin reduction (2 to 4 g/dL) was observed in three patients, and intestinal thrombosis was recorded in one patient. The study did not show the impact of the SARS-CoV-2 spike protein on hemolysis [12]. The literature contains a description of a case of severe hemolysis in an 81-year-old woman after the second dose of the Moderna vaccine, which was then complicated by acute kidney injury [13]. The overview by Fattizzo B. et al. (2021) presented eight cases of hemolysis in patients with PNH after mRNA-based vaccines against SARS-CoV-2 (Pfizer, Moderna) [11].

A retrospective, observational, cohort, non-comparative study of the features of the course of COVID-19 in patients with PNH was performed to assess the specific features of COVID-19 in patients treated with eculizumab, as well as the impact of COVID-19 on vaccinated and non-vaccinated patients with PNH.

## 2. Methods

### 2.1. Patient Population

The study included 52 patients with PNH, followed up in the Moscow City Hematology Center (MCHC) and eligible for anti-complement treatment for the underlying disease. MCHC is the largest city hematology center in Russia. MCHC is the base for the management of patients with orphan diseases in Moscow, and one of the largest research centers for the treatment of orphan diseases in the Russian Federation. Thirty-eight (72%) of these patients received background therapy with eculizumab (Elizaria), and the others were on anti-complement treatment in clinical studies. Eculizumab (Soliris) began being used for treatment in 2012, and treatment with the biosimilar Elizaria was initiated in 2019. 

The study included patients aged 18 to 81 years and diagnosed with PNH; they had confirmed flow cytometry results according to the PNH clone size in RBCs and WBCs, intravascular hemolysis, and concomitant clinical symptoms at the time of presentation to the center (or in their history) and were all treated for PNH with eculizumab or ravulizumab. At the time of participation in the study, the patients had all been vaccinated and revaccinated against meningococcal infection (serotypes A, C, Y, and W135 Neisseria meningitidis) within less than 5 years. Exclusion criteria were active Neisseria meningitidis infection during follow-up, a history of allergic reactions to eculizumab, severe somatic or mental disorders, and pregnancy or breastfeeding. 

Patients were divided into two groups for comparison of the incidence of COVID-19. Group 1 included non-vaccinated patients with PNH, and Group 2 included vaccinated patients prior to the onset of COVID-19. According to vaccination, patients were subdivided into vaccinated and non-vaccinated groups. The group of vaccinated patients included patients who were vaccinated without signs of past COVID-19 at the beginning of analyzed period, and patients vaccinated half a year or more after recovery from COVID-19.

### 2.2. Study Design

A retrospective, observational, cohort, non-comparative, study was performed to assess the course of COVID-19 as well as the safety and efficacy of the Gam-COVID-Vac vaccine at patients with paroxysmal nocturnal hemoglobinuria. The study was approved by the Independent Interuniversity Ethics Committee (Extract from Minutes No. 06, dated 16 June 2022) and was conducted in accordance with the ethical standards of the responsible committees and with the Helsinki Declaration of 1975, as revised in 2013.

The observation period included the COVID-19 pandemic time between March 2020 and January 2022. In this period, all patients with PNH in the MCHC were under close monitoring and each patient visited the center 3–4 times a month.

### 2.3. Treatment and Vaccination

Eculizumab-naive patients who started treatment during the study period received an initial cycle of therapy consisting of four weekly intravenous administrations of the the complement inhibitor at a dose of 600 mg, with subsequent maintenance therapy at a dose of 900 mg every two weeks. Patients previously treated with eculizumab received it at a maintenance dose of 900 mg every two weeks.

Gam-COVID-Vac is an adenovirus viral vector vaccine that comes in two doses (components) 21 days apart. Each dose was based on one of two human adenoviruses: Ad26 (serotype 26) or Ad5 (serotype 5). The viruses contain the gene that encodes the full-length spike protein (S) of SARS-CoV-2. The vaccine was administered intramuscularly; the initial component I was given at a dose of 0.5 mL and, after 3 weeks, component II was given at a dose of 0.5 mL.

### 2.4. Investigations

COVID-19 was diagnosed according to the results of PCR testing, which was carried out by the Moscow Outpatient Service in cases of hyperthermia or other symptoms of this disease, and on a routine basis prior to target therapy administration. Testing for anti-SARS-CoV-2 IgG was carried out monthly during the year after the episode of COVID-19 in patients with PNH to assess the immune status dynamics, as well as to decide on vaccination or revaccination. Testing for anti-SARS-CoV-2 IgG levels in the blood serum was carried out in the local laboratory of the S.P. Botkin City Clinical Hospital using the semi-quantitative method featuring a chemiluminescence immunoassay (CLIA). A test for anti-SARS-CoV-2 RBD IgG levels was additionally performed on vaccinated patients to assess the immune status. Blood sampling for anti-SARS-CoV-2 RBD IgG in vaccinated patients was carried out in the interval from 2 to 9 months after vaccination. Tests for anti-SARS-CoV-2 RBD IgG levels were carried out in the InVitro Laboratory using the quantitative method in the blood serum, featuring a chemiluminescent immunoassay analysis (Architect technology, Abbott. SARS-CoV-2 IgG II Quant). The test for anti-SARS-CoV-2 IgG titers was carried out once a month for 12 months after COVID-19. Vaccination of patients with PNH with Gam-COVID-Vac (Sputnik V) was conducted according to the prescribing information. The patients treated with eculizumab were vaccinated within 2 to 7 days after administration of the target agent. 

### 2.5. Endpoints

The endpoints for evaluating the results of vaccination against COVID-19 in patients with PNH included frequency of hospitalizations with COVID-19 among patients with paroxysmal nocturnal hemoglobinuria; the proportion of patients with paroxysmal nocturnal hemoglobinuria who did not develop COVID-19 after vaccination; the titer of immunoglobulins G (IgG) in patients with paroxysmal nocturnal hemoglobinuria after COVID-19; the titer of IgG to the RBD domain in patients with paroxysmal nocturnal hemoglobinuria vaccinated and non-vaccinated against COVID-19; and the titer of IgG to the RBD domain in patients with paroxysmal nocturnal hemoglobinuria who are treated with eculizumab and with another therapy.

The endpoints for evaluating the results of targeted treatment among patients vaccinated with the Gam-COVID-Vac vaccine and patients with COVID-19 included the incidence of breakthrough hemolysis in patients with paroxysmal nocturnal hemoglobinuria due to COVID-19; the need for blood transfusions in patients with paroxysmal nocturnal hemoglobinuria due to COVID-19; and the need to change the dose or mode of administration of eculizumab in the treatment of paroxysmal nocturnal hemoglobinuria due to COVID-19. 

The safety endpoints in the study were the development of adverse events associated with vaccination for the prevention of COVID-19; development of allergic reactions; and the development of adverse events associated with targeted therapy aiming to inhibit the complement system.

### 2.6. Statistical Analysis

A statistical analysis was performed using SPSS, version 17.0. Descriptive statistics were used for demographics, baseline parameters, and parameter values at all the time points of the study. Demographics were described for all the included patients. The safety analysis included all patients who had PNH and were under follow-up. An analysis of target treatment and anti-COVID-19 vaccination parameters was carried out on all the patients included in the study who provided their demographic data. Demographics, baseline parameters, parameter values during treatment period, and changes from baseline were tabulated using descriptive non-parametric statistics (mean, standard deviation, median, minimum and maximum values, range, quartiles, number of valid cases for quantitative variables, and number, percentage, and distribution for dichotomous and qualitative variables). Paired statistical comparisons in the group were made using a nonparametric Pearson’s chi-squared test or a Wilcoxon signed-rank test for matched pairs to analyze changes in the safety parameters at study visits compared to screening.

## 3. Results

The investigators screened 52 patients, all of whom met all the inclusion/exclusion criteria and were enrolled in the study. The median age of the enrolled patients was 31.0 (18.0–81). Among them were 9 (17%) males and 43 (83%) females. The general characteristics of the patients are given in Table 1.

Since 2019, 38 patients had been treated with Elizaria, 27 (71%) of whom were switched from Soliris, and 11 (29%) patients received only Elizaria. Between March 2020 and January 2022, positive PCR test results were identified in 28 (53.8%) enrolled patients, during a routine examination before the administration of the target agent. Among them, 16 (57%) patients had mild forms of COVID-19, and 7 (25%) patients had asymptomatic forms. Five (18%) patients required hospitalization, only one (3.5%) of whom was vaccinated against COVID-19 two months prior to the positive PCR test result. The other four (14%) patients with severe forms of COVID-19 were non-vaccinated. All these patients had PNH clones ≥50%. Among them, three (11%) male patients were aged 60–70, and two (7%) female patients were aged 30–40. One (3.5%) patient had regular blood transfusions due to an insufficient hematological response to targeted therapy. Breakthrough hemolysis and severe lung damage according to CT data (moderate-to-severe pneumonia, with lung damage of 50–75%) were reported in each of these hospitalized patients. Moreover, all the patients had to resume hemotransfusions. None of the patients needed artificial lung ventilation (ALV); no fatal outcomes were reported.

Some 22 (42.3%) of the 52 patients (19 of them with a hemolytic variant of PNH) were fully vaccinated with Sputnik V. Of these 22, 25% were vaccinated without signs of past COVID-19 at the beginning of the analyzed period, and 9 (17.3%) out of the 22 patients were vaccinated half a year or more after recovery from COVID-19 to prevent the development of a repeated case.

**Figure 1:**
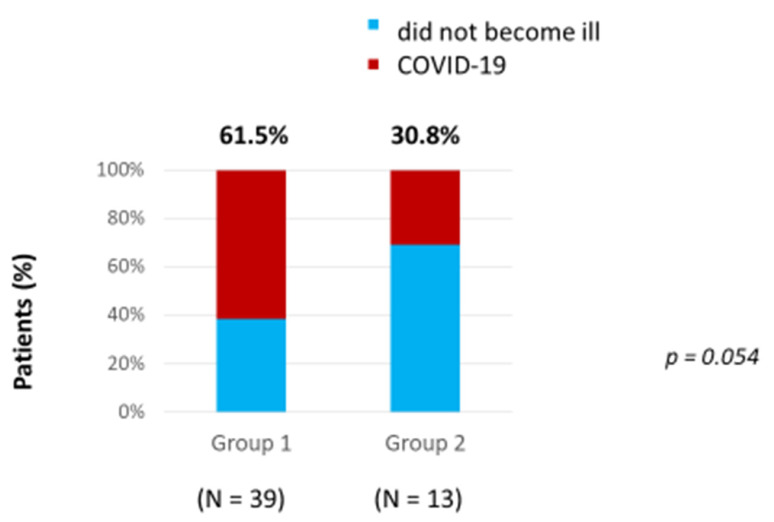
Incidence of COVID-19 in vaccinated and non-vaccinated patients.

A comparison of the incidence of COVID-19 was carried out between 39 non-vaccinated patients with PNH (Group 1) and 13 vaccinated patients (Group 2) prior to the onset of COVID-19. The number of patients who had COVID-19 was about two times higher in the Group 1 of non-vaccinated patients, and amounted to 24 (61.5%), while in Group 2, which included vaccinated patients, the number of patients with COVID-19 was only 4 (30.8%) (Figure 1). Due to this, the number and proportion of patients who had no COVID-19 was higher in Group 2, which included vaccinated patients, at 9 (69.2%), than in Group 1 with non-vaccinated patients, which had 15 (38.5%) An efficacy analysis of vaccination with Sputnik V using the Pearson’s chi-squared test demonstrated that this number of patients without COVID-19 in Group 2 was very close to being statistically higher than in Group 1 with non-vaccinated patients (*p* = 0.054) (Figure 1). However, due to the small number of patients, a statistical difference was not determined. In Group 1, nine patients (17.3%) were vaccinated after suffering with COVID-19, in order to prevent the development of a repeated case.

**Figure 2:**
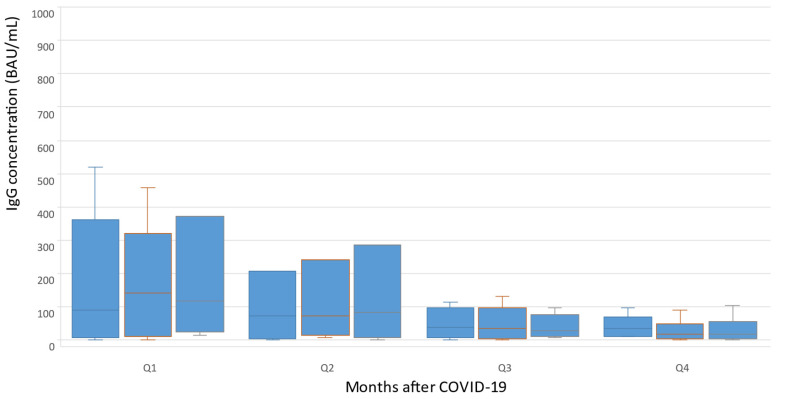
Changes in concentrations of anti-SARS-CoV-2 IgG during the year after COVID-19.

An analysis of changes in the concentrations of anti-SARS-CoV-2 IgG was carried out during the year after the episode of COVID-19 in the group of patients with PNH to assess their immune status in dynamics, as well as decide on vaccination or revaccination. The results showed that the peak titer of anti-SARS-CoV-2 IgG was revealed 2–3 months after the acute infection phase 127 [16; 276], using BAU/mL, in the first quarter of the annual observation (Q1). In the second quarter (Q2), the titer of anti-SARS-CoV-2 IgG decreased to 58 [17; 87] BAU/mL after 4 months, and then tended to increase gradually by 6 months after patients had recovered. In the third quarter of the annual observation (Q3), the titer of anti-SARS-CoV-2 IgG decreased again to 28 [8; 83] BAU\ml by the 7th month, and then remained at a consistently low level. In the fourth quarter (Q4), there was a subsequent gradual decrease in the titer of anti-SARS-CoV-2 IgG to 23 [4; 36] BAU/mL by month 11–12 (Figure 2). 

**Figure 3:**
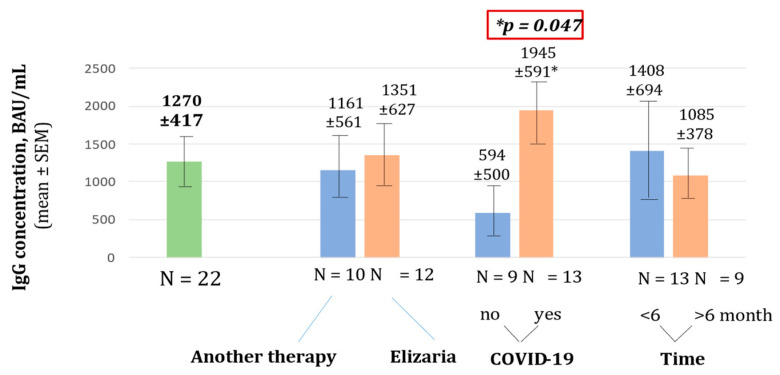
Concentration of IgG for RBD SARS-CoV-2 spike (S) protein.

In December 2021, testing for anti-SARS-CoV-2 RBD IgG levels was performed in 22 patients. Blood sampling was carried out in the interval from 2 to 9 months after vaccination. The IgG for RBD SARS-CoV-2 spike (S) protein concentration in whole group was relatively high and amounted to 1270 ± 417 BAU/mL. Patients after COVID-19 showed a statistically significant increase in their immunoglobulin titers; the mean IgG for RBD SARS-CoV-2 spike (S) protein concentration was higher in the group of patients after suffering with COVID-19—1945 ± 591 BAU/mL, than in the group of patients without COVID-19—594 ± 500 BAU/mL (*p* = 0.047). Factors such as therapy, type including eculizumab, and time from disease onset of more or less than 6 months, did not have a significant impact on antibody titers (*p* > 0.05). Notably, the treatment with eculizumab demonstrated no effect on antibody titers (Figure 3). Patients treated with eculizumab had IgG for RBD SARS-CoV-2 spike (S) protein concentration of 1351 ± 591 BAU/mL and patients treated without eculizumab had an IgG concentration of 1161 ± 561 BAU/mL. Patients vaccinated less than 6 months prior had a non-significantly higher concentration of IgG for RBD SARS-CoV-2 spike (S) protein −1408 ± 694 BAU/mL, while patients vaccinated for more than 6 months had a smaller concentration of 1085 ± 378 BAU/mL.

In patients treated with eculizumab, the overall clinical response according to hemoglobin level was achieved in 28 (74%) patients. Hemoglobin levels >110 g/L were found in 11 (29%) patients, 110–80 g/L in 17 (45%); 10 (26%) in patients receiving regular hemotransfusions, and 4 (40%) had anemia aplastic.

Both first and second doses of the vaccine were well tolerated; all reported AEs were classified as grade 1–2 according to CTCAE v5.0 [14]. After the first dose, AEs were reported in nine (41%) patients, including four (18%) cases of body temperature increases, three (13.6%) cases of headache, one (4.5%) case of pain in joints, and one (4.5%) case of colitis exacerbation. Pain in joints and colitis exacerbation occurred in a PNH patient with multiple comorbidities (rheumatoid arthritis, nonspecific ulcerative colitis, systemic lupus erythematosus). After the second dose, no AEs were reported. Vaccine administration was not accompanied by pharmacodynamic breakthrough hemolysis or hemolysis exacerbation; it did not require hospitalization either.

## 4. Discussion

In this study, an assessment of the impact of COVID-19 on vaccinated and non-vaccinated patients with PNH was carried out to evaluate the efficacy and the Gam-COVID-Vac vaccination. Additionally, the specific features of COVID-19 in patients treated with eculizumab were studied. The study was conducted during the pandemic from March 2020 to January 2022, so the effectiveness of vaccination may have changed due to changing strains of COVID-19. Particular attention was drawn to the emergence of the “Omicron” strain and its varieties in late 2021–early 2022, in which the vaccination efficiency was lower than with earlier strains, which led to the search for ways to optimize vaccination [15]. The study demonstrated that the number of patients who had COVID-19 was about two times higher in the group of non-vaccinated patients. Thus, a two-fold reduction in the incidence of COVID-19 in patients with PNH vaccinated with Gam-COVID-Vac was found. Hence, vaccination in patients with PNH with the Gam-COVID-Vac may be effective for COVID-19 prevention. This result of vaccine efficacy is slightly less than in the general population [16]. However, it must be taken into account that the majority of patients with PNH received complement blocking therapy, and in some cases had bone marrow failure.

COVID-19 and, in some cases, vaccines against this infection can trigger disease aggravation in patients with PNH. Kamura Y. et al. (2022) described the development of pronounced hemoglobinuria after administration of the BNT162b2 vaccine (Pfizer/BioNTech) and mRNA-1273 vaccine (Moderna) in 2/5 patients with PNH who did not receive target therapy and in 1/12 patients treated with complement inhibitors [17]. In the study conducted by Gerber G.F. et al. (2021), hematuria and breakthrough hemolysis were reported in four out of six patients after the administration of both the first and second components of the vaccine [12]. An Italian multicenter study showed that hemolytic events after vaccination against SARS-CoV-2 were observed in approximately 6% of patients [18]. In our study, no cases of underlying disease aggravation were reported after vaccination. AEs occurred in 40% patients, but all of them were mild or moderate and did not require additional interventions. These differences may be due to the different vaccines that were used for vaccination. Additional studies are required to clarify the effect of each vaccine on the activation of the complement system.

The literature contains single descriptions of the specific features of the course of COVID-19 in patients with PNH. According to Fattizzo B. et al. (2021), 89.4% of patients have mild and moderate-to-severe forms of infection, which is consistent with the data obtained in our study. The complement system is likely to participate in the pathogenesis of COVID-19, including in the development of respiratory failure, intravascular coagulopathy, and pronounced inflammation. It was also noted that complement inhibitors may suppress excessive inflammatory activity caused by SARS-CoV-2 [11]. On the other hand, patients with PNH are at risk of developing breakthrough hemolysis whilst receiving target therapy, which may be due to the increased activity of the complement system caused by COVID-19 [19].

The conducted study demonstrated that, in some cases, SARS-CoV-2 infection led to PNH aggravation, although the majority of patients receiving anticomplementary therapy had mild or asymptomatic forms of COVID-19, with an increased risk of breakthrough hemolysis. In the study, this complication was reported only in five (18%) patients. To prevent the aggravation of PNH, patients with confirmed positive or suspicious results of SARS-CoV-2 testing should strictly adhere to intervals between injections of target agents.

The conducted study has several limitations, including the retrospective character of its data collection, and the small number of patients under follow-up. In spite of this, the obtained results confirm evidence from earlier studies [11,12,17,18].

## 5. Conclusions

The study demonstrated a two-fold reduction in the incidence of COVID-19 in patients with PNH who were vaccinated with Gam-COVID-Vac (Sputnik V), compared with those not vaccinated. Moreover, the administration of the first and second components was characterized by good and excellent tolerability, respectively, since they were not accompanied by outbreaks of breakthrough hemolysis or serious adverse events. These results suggest that Gam-COVID-Vac (Sputnik V) for COVID-19 prophylaxis is effective and safe in patients with PNH and immunosuppression who receive target therapy. However, further larger randomized trials are required in patients with PNH.

## Figures and Tables

**Table 1:** General characteristics of the study patients.

Parameters	Patients Treated with Elizaria, *n = 38*	Patients on Any Anti-Complement Treatment, *n = 52*
Age at PNH diagnosis, years, median (min–max)	31.5 (18.0–81)	31.0 (18.0–81)
Males, %	12	17
Females, %	88	83
Time from the start of PNH diagnostics to the start of therapy, months, median (Q25; Q75)	14.8 [5.9; 35.0]	13.5 [4.1; 37.2]
Age during therapy with Elizaria, mean (min–max) years	47.3 (18.0–81)	–
Clone ≥50%, %	37 (97%)	51 (98%)
Clone ≤10%, %	1 (3%)	1 (2%)
Therapy duration, month, median (Q25; Q75)	57.6 [30.0; 73.5]	48.1 [27.7; 73.5]

## Data Availability

The data are not available in the public domain, but they may be available upon request from the corresponding author after approval from the principal investigator.
